# Leech Therapy for Treating Priapism: Case Report

**Published:** 2017-07

**Authors:** Sayed Aladdin ASGARI, Sadeq ROSTAMI, Mojtaba TEIMOORI

**Affiliations:** 1. Urology Research Center, Razi Hospital, School of Medicine, Guilan University of Medical Sciences, Rasht, Iran; 2. Dept. of Urology, Guilan University of Medical Sciences, Rasht, Iran

**Keywords:** Priapism, Leeches, Treatment

## Abstract

Priapism is well-defined by persistent, painful penile erection which happens without sexual stimulation. Currently, the hirudotherapy is practiced to treat venous congestion and subsequent compartment syndrome. Here we will report a case of a male with priapism treated by leeches. The case was a 26 yr old young single male referred to the Razi Hospital Emergency Department, Guilan University of Medical Sciences, Rasht, Iran due to long-time spontaneous erections. The patient had no history of mental disorders, trauma or sickle cell anemia. we insert two leeches in each side of penile shaft for two hours, after a one hour break we insert do in same manner for another cycle. At follow-up two days later he had significantly decreased pain, though still had cavernosal swelling and tenderness to palpation. The patient was subsequently discharged after three days of admission. The pain and perineal swelling completely resolved over the course of one month. In this case, chronology indicates that leech therapy was possibly treatment option for priapism. This procedure seems to be non-invasive treatment strategy worth to discussing in such patients.

## Introduction

Priapism is a continued erection without sexual stimulus. Priapism has some great consequence, a more common and most important is erectile function. It has a great emotional, socioeconomic, and physical aspect. Ischemic, non-ischemic, and stuttering are different types of priapism. In ischemic priapism, treatments are emergent because ischemia can result in fibrosis and erectile dysfunction later (1).

Present approach to ischemic priapism is early corporeal aspiration, irrigation, and injection of intracavernosal phenylephrine. Surgical methods after that are very different from T-shunts in corpus cavernous, shunts in proximal corpus spongiosum, or distal shunt. It is declared without intervention after twenty-four-hour erectile dysfunction will occur (2).

Leech therapy had old history and after that time medicinal leech practiced for many medical situations, now, hirudotherapy come back in some clinical condition (3). A painting far away in history shows the use of leeches in medicine (4, 5). Their practice has wide-ranging changes during the history of 3500 yr, through treatments extending in vast majority of disease (6). Gradual bleeding in controlled condition can cause purification of blood and healing disease (5). Leech therapy was the source of the effective practice until the beginning of last century (7). In the 1880s, leech’s saliva antithrombotic factors were found and revealed the anticoagulant properties and termed it hirudin in early 19^th^ century (8). In 1960, re-attention in hirudotherapy by plastic surgeons commenced by means of leeches to treat venous congestion (9). Condemnation to leech therapy waned in the late nineteenth and early twentieth century, but scientific interest in leech in medicine sustained (10, 11).

Leeches are extremely valuable in medicine (12). Currently, the hirudotherapy is practiced to treat venous congestion and subsequent compartment syndrome in reconstructive surgery, traumatology (13, 14). Although through times, it settled a common therapeutic approach for numerous diseases but failed after the mid-nineteenth century due to the growth of new medicine. In 2004, this treatment process received the approval of the Food and Drug Administration (FDA) in the USA (10), European leech therapy is recycled in medicine by a new term of hirudotherapy.

### Case presentation

A 26-yr-old man presented to the Razi Hospital Emergency Department, Guilan University of Medical Sciences, Rasht, Iran with severe, penile pain with erection of 80 h duration. He described the pain as non-radiating and sharp, denied any trauma, without any lower urinary tract symptoms. The patient was single and had no history of psychiatric drug consumption. Physical examination was remarkable a tender, firm erected penis. Complete blood count, comprehensive metabolic panel, and urinalysis were unremarkable, that all were normal.

Color Doppler sonography confirmed ischemic priapism. In our settings, we did not put any penile prosthesis, due to non-availability of an immediate prosthesis and financial constraints with the patients.

After we obtained informed consent form patient, we protected a glans and urethra by a tape. The patient antimicrobial prophylaxis was with intravenous Cephtriaxone (1.5 gr twice daily) and antihistamines for 7 d.

**Fig.1:**
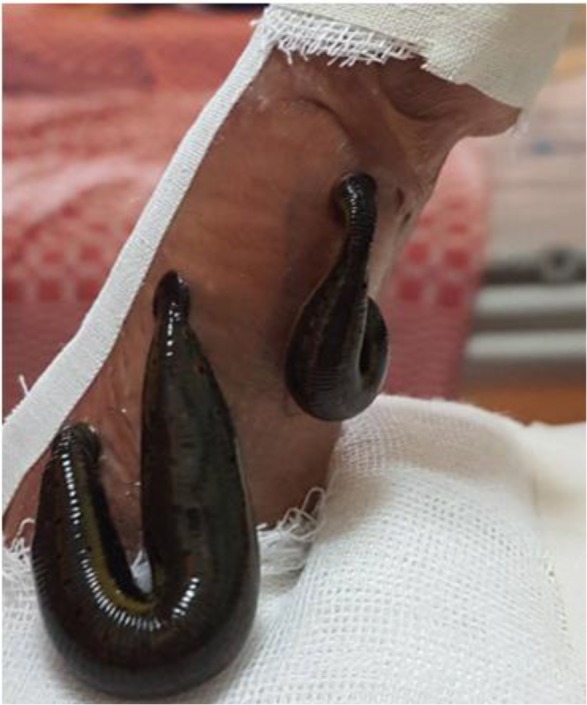
A protection a glance and urethra by tape and two leeches in every side of penile skin shaft

We applied two leeches (from hirudo medicinal family) on each side of penile shaft, we adhered to feed until engorged, at this point they willingly detached, after a one hour break we insert do in same manner for another cycle (Fig. 1).

We had patient under close observation and coagulation test was done frequently during leech therapy. At follow-up two days later he had significantly decreased pain, though still had cavernosal swelling and tenderness to palpation. The patient was subsequently discharged after three days of admission.

The pain and perineal swelling completely resolved over the course of one month. At first follow-up one month after initial presentation, the patient reported continued resolution of penile pain, mild to moderate impairment of erectile function that responded to tadalafil and he experienced no further episodes of priapism.

## Discussion

In a patient with Priapism after twenty-four hours, penile prosthesis placement must be considered and patient consultation about it seems to be essential, and if patient refuse, poor erectile function is expected (15).

Leeches are usually used when venous drainage is not equal to the arterial arrival, this condition may result in venous congestion, apoptosis may occur. Hirudotherapy is used to save circulation by creating temporary venous drainage until permanent drainage made (16). The accomplishment of non-microsurgical replantation possibly will be one of the less common practices of hirudotherapy (16), and maybe hirudotherapy become new chapter in urology era.

Vascular congestion because of compartment syndrome and later ischemia bring an important condition in therapeutic procedures that reveal necessity for hirudotherapy (17). Priapism also can have considered exaggerated blood congestion but in the presence of loss of arterial inflow. There are past reports about urologic trauma and penile amputation and its challenges (14, 18), and showed venous drainage is serious to the success of replantation (18).

Pain relief in our patient soon after hirudotherapy is due to the being of anti-inflammatory factors in the saliva of leeches. Hirudotherapy has also practiced in general and localized inflammation and pain treatment. Leech saliva has vigorous elements with anti-inflammatory, effect. An exact analgesic factor in the leech saliva is, however, to be recognized. Pain relief after leech therapy is fast, effective and long-lasting (19, 20).

One of the most hirudotherapy considerations is complication, most dangerous complications local infections with, especially *Aeromonas* spp. until now, ranging from superficial to deep infections like cellulitis and subcutaneous abscess (21) to ultimately septicemia (22). In case of resistance to antibiotics, prescribing fluoroquinolones is proposed (23).

The patient maybe refuses such treatment or terminates treatment among the procedure because of psychological aspect of such treatment. Sustained and uncontrolled leech apply may result in bleeding and anemia. Allergic local responses and anaphylaxis also were reported (24).

Regarding the choosing hygienic leech and prophylactic antibiotic therapy and close patient observation, we had no short-term complication and 30 days after procedures.

The authors of this case report concur with the published criticism of the initial report - undertaking the risks associated with non-operative approach to late priapism are currently not justified. Nevertheless, for the sake of taking a step ahead in science, we report this.

## Ethical considerations

Ethical issues (Including plagiarism, informed consent, misconduct, data fabrication and/or falsification, double publication and/or submission, redundancy, etc.) have been completely observed by the authors.
